# Clostridium Perfringens as a Rare Cause of Spontaneous Bacterial Peritonitis

**DOI:** 10.7759/cureus.68363

**Published:** 2024-09-01

**Authors:** Javier E Canahuati Escobar, Dean Hasan, Roy L Dennis, Mohamed Faris

**Affiliations:** 1 Internal Medicine, Grand Strand Medical Center, Myrtle Beach, USA; 2 Internal Medicine, Edward Via College of Osteopathic Medicine (VCOM), Myrtle Beach, USA; 3 Internal Medicine, Edward Via College of Osteopathic Medicine (VCOM) Carolinas, Myrtle Beach, USA

**Keywords:** prophylactic antibiotics, ascites, gram positive bacteria, spontaneous bacterial peritonitis, clostridium perfringens

## Abstract

Spontaneous bacterial peritonitis (SBP) is a serious complication in individuals with liver cirrhosis and ascites. In this case report, we present an unusual cause of SBP in loculated ascites caused by an uncommon bacterium, *Clostridium perfringens*. Although SBP is typically associated with certain common pathogens, it is important to recognize that less frequent organisms can also trigger it. *C. perfringens* is typically associated with other environmental sources, but in this instance, the infection’s origin was suspected to be either nosocomial, from prior paracentesis, or due to a microscopic bowel perforation that was undetectable on imaging. Remarkably, the patient responded well with an improvement of symptoms, and the ascitic fluid bacterial growth resolved on subsequent cultures.

## Introduction

Spontaneous bacterial peritonitis (SBP) is a potentially life-threatening infection characterized by the spontaneous inflammation of the peritoneum, the lining of the abdominal cavity, without an evident source of infection. This condition predominantly occurs in individuals with advanced liver disease, particularly those with cirrhosis and ascites (accumulation of fluid in the abdominal cavity).

SBP is caused by the translocation of bacteria from the intestines into the ascitic fluid, leading to infection. Commonly implicated bacteria include *Escherichia coli*, *Klebsiella pneumoniae*, and various streptococcal species. The condition is diagnosed through the analysis of ascitic fluid obtained via paracentesis, with a polymorphonuclear leukocyte (PMN) count greater than 250 cells/mm³ being a key diagnostic criterion [1].

Clinically, patients with SBP may present with a range of symptoms including fever, abdominal pain or tenderness, altered mental status, and signs of systemic inflammation. However, symptoms can be subtle or nonspecific, particularly in those with advanced liver disease, making a high index of suspicion critical for early diagnosis.

The management of SBP involves prompt antibiotic therapy, typically with third-generation cephalosporins such as cefotaxime, to cover the common causative organisms. Prophylactic antibiotics may also be administered to high-risk patients to prevent recurrence [1,2]. Additionally, addressing the underlying liver disease and managing ascites are essential components of the long-term treatment strategy.

Despite advancements in diagnosis and treatment, SBP remains a significant complication of cirrhosis with a high morbidity and mortality rate, underscoring the importance of early recognition and prompt intervention to improve patient outcomes.

This case underscores the critical importance of early diagnosis, appropriate antibiotic therapy, and the consideration of unusual pathogens in patients with cirrhosis and ascites. Clinicians must maintain heightened vigilance for SBP, especially in those with significant risk factors such as alcohol-related liver disease and a history of invasive procedures like paracentesis [3]. Early intervention and tailored treatment strategies are vital for improving patient outcomes and preventing complications associated with this serious condition. Additionally, regular monitoring and follow-up care are essential to ensure timely detection and management of any recurrent or new infections [2].

## Case presentation

Our patient was a 63-year-old male with a past medical history of alcoholic cirrhosis with recurrent ascites requiring large-volume paracentesis, hypertension, hepatitis C, type 2 diabetes mellitus, alcohol-related chronic pancreatitis, hip arthroplasty, and deep vein thrombosis for which he was taking apixaban. He presented to the hospital multiple times due to shock, respiratory failure, and recurrent ascites.

The patient initially presented to the emergency department with a chief complaint of abdominal pain and distension for three days and sought evaluation following the advice of his home health provider. Additionally, he complained of swelling and edema in his lower extremities, similar to when he was diagnosed with deep vein thrombosis earlier that year. The patient was admitted and underwent an ultrasound (US)-guided paracentesis with 6 L removed in addition to a thoracentesis with 2 L removed. Albumin was given according to replacement guidelines per standard of care when performing large-volume paracentesis. He was then discharged home on spironolactone and furosemide.

Shortly after his discharge, he returned to the hospital via Emergency Medical Services (EMS) with complaints of acute-onset shortness of breath, lower back pain, and abdominal pain. He also endorsed lightheadedness and intermittent blurry vision. The patient stated that the fluid reaccumulated faster than usual following his discharge. Repeat paracentesis was performed while the patient was admitted and 3.1 L of fluid was removed. The results of the paracentesis and thoracentesis revealed SBP criteria with neutrophil count (PMNs) of 5917.

**Figure 1:**
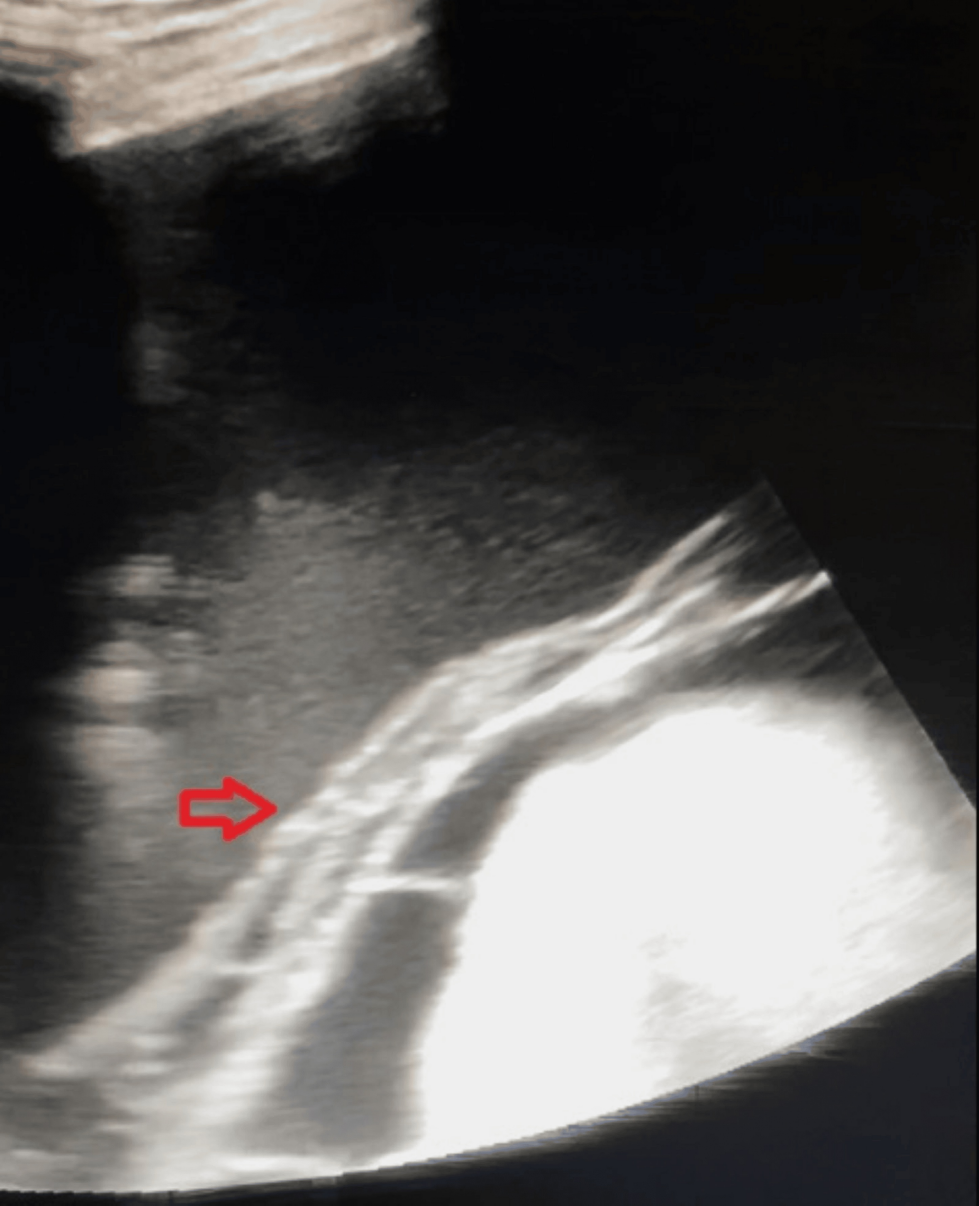
Initial ultrasound findings before the first paracentesis. Findings were consistent with mild loculated ascites and fibrinous strands (red arrow) forming multiple loculations.

**Figure 2:**
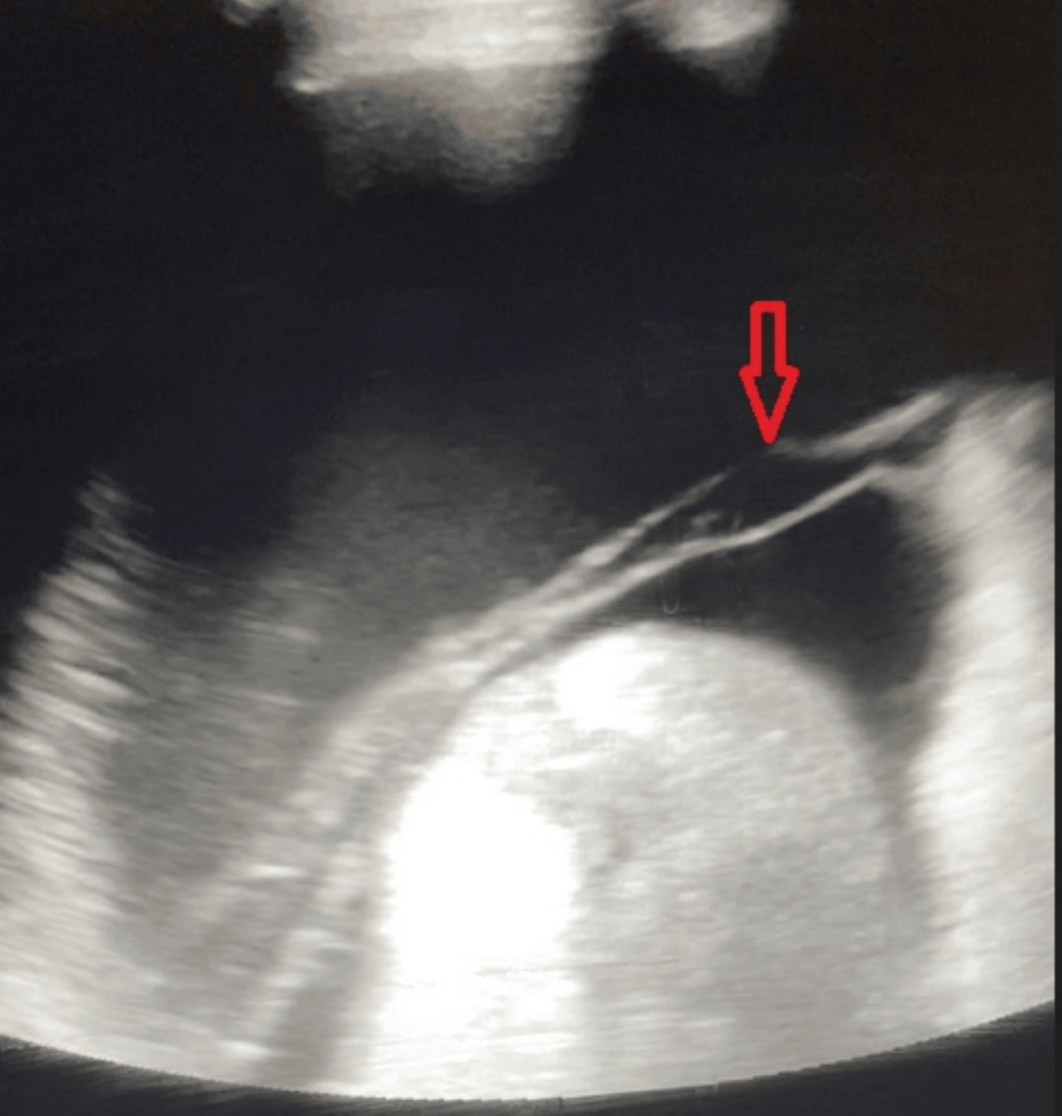
Initial ultrasound findings before the first paracentesis. Findings were consistent with mild loculated ascites and fibrinous strands forming multiple loculations (red arrow).

The patient was admitted to the hospital due to septic shock secondary to suspected bacterial peritonitis. Initial management included the administration of 2 grams of ceftriaxone, fluid resuscitation, computed tomography (CT) imaging, and diagnostic paracentesis. The findings from these procedures are illustrated in Figures 1, 2.

The initial CT scan revealed liver cirrhosis with large-volume ascites. The patient's model for end-stage liver disease (MELD) score was 25, indicating severe liver dysfunction, and the patient was classified as Child-Pugh class B. Diagnostic paracentesis showed a polymorphonuclear leukocyte (PMN) count exceeding 5000 cells/mm³. Ascitic fluid cultures grew *Clostridium perfringens*, resistant to clindamycin. In response, an infectious disease specialist was consulted and recommended the addition of metronidazole to the antibiotic regimen.

**Figure 3:**
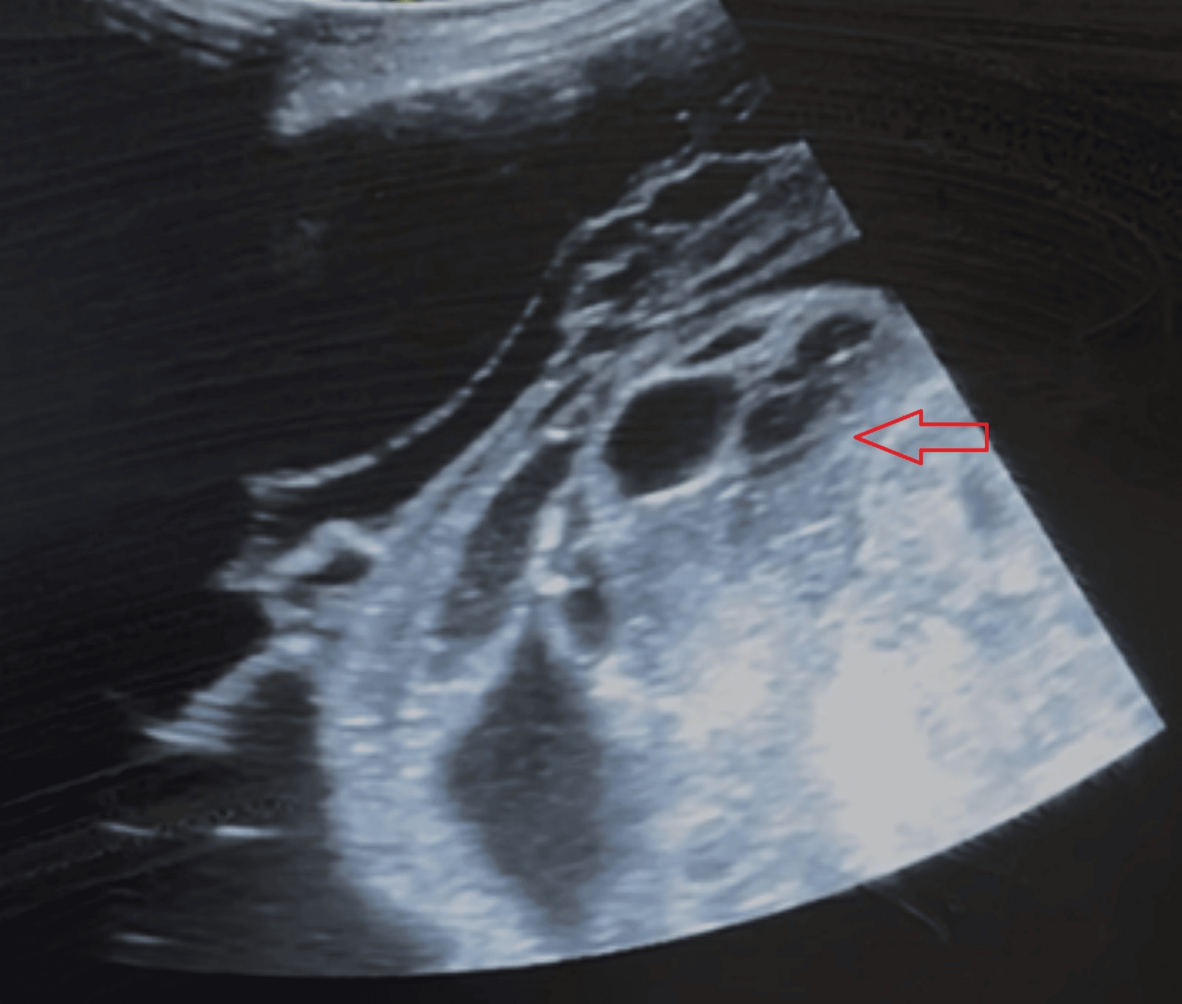
Increased number of loculations (red arrow) with thickened fibrinous walls observed 14 days later, despite the initial antibiotic treatment

**Figure 4:**
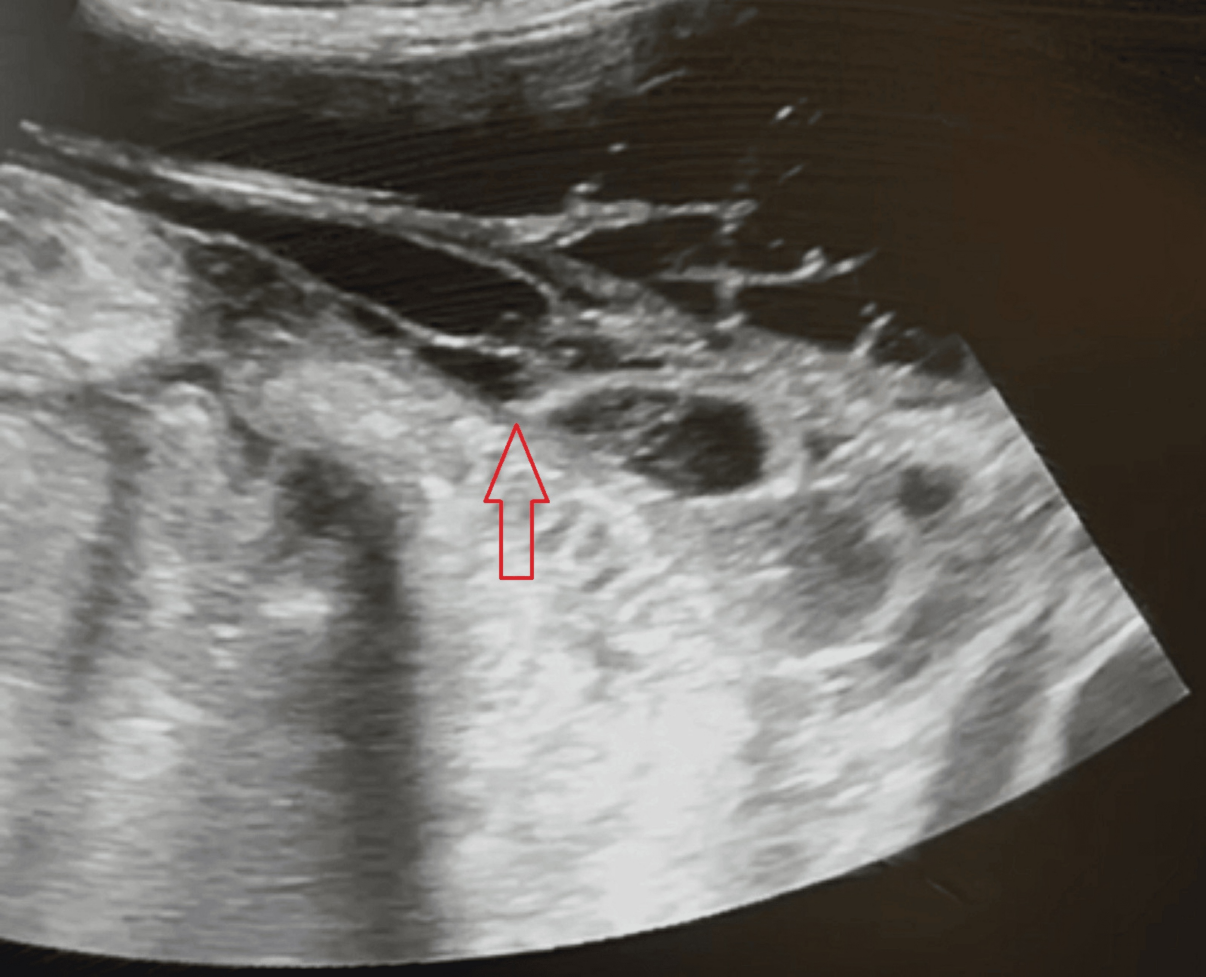
Increased number of loculations with thickened fibrinous walls (red arrow) observed 14 days later, despite the initial antibiotic treatment

The patient's condition initially improved with this modified treatment approach. However, two weeks later, the patient's status deteriorated again. Despite ongoing antibiotic therapy and multiple therapeutic paracentesis, further complications arose such as episodic pain, tachycardia, reaccumulation of fluid, and increased number of loculation, as depicted in Figures 3, 4.

**Figure 5:**
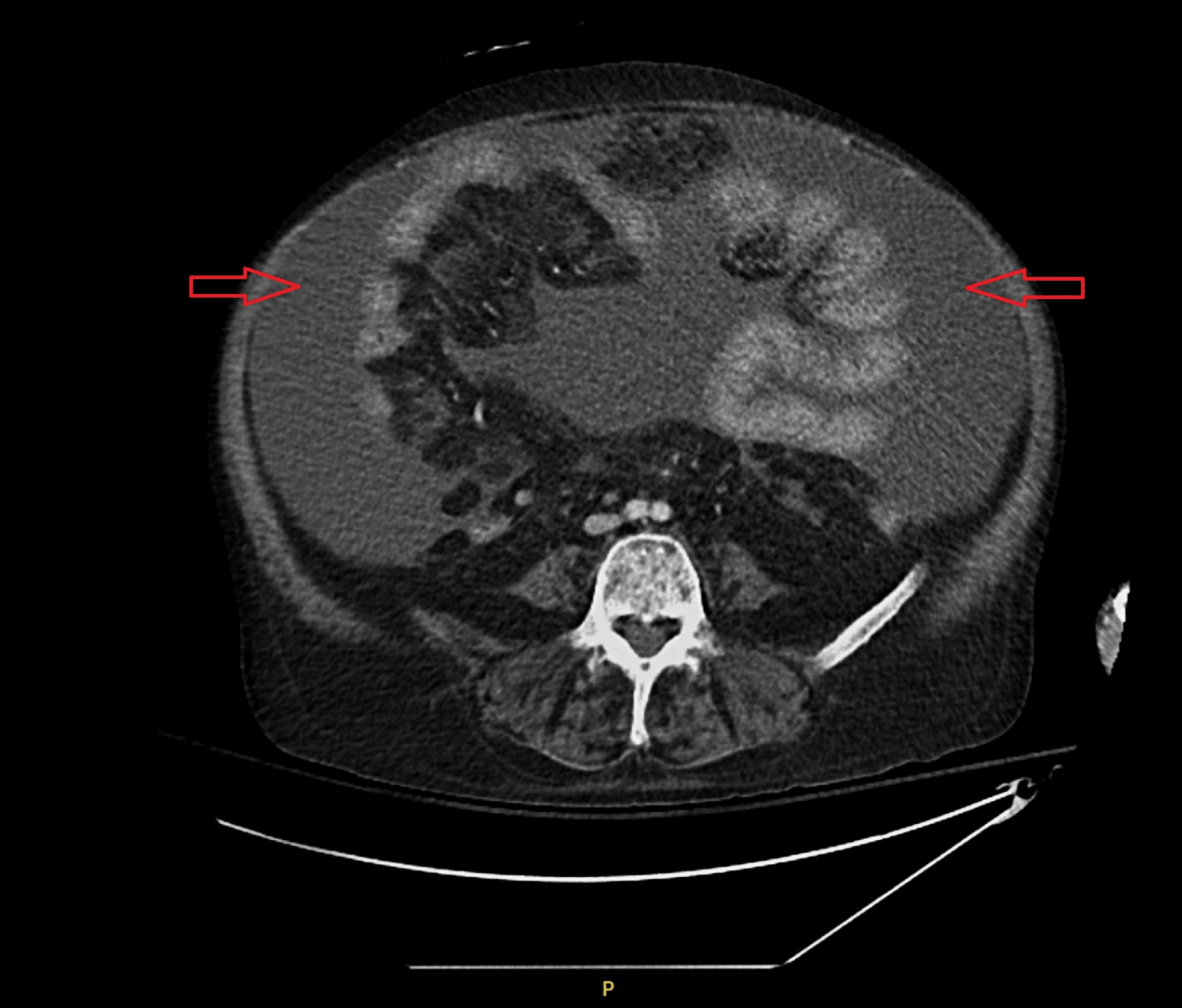
CT scan of the abdomen with moderate volume ascites (red arrows), morphologic changes of cirrhosis, and without evidence of pneumoperitoneum

A repeat CT scan did not reveal gastrointestinal perforation as seen in Figure 5, and subsequent cultures failed to grow any organisms.

Given the patient's progressive decline and lack of response to treatment, goals of care conversations were held to discuss the patient's prognosis and care options. Ultimately, the patient and their family decided to transition to comfort care. The patient was discharged home with hospice care to focus on the quality of life and symptom management during the final stages of illness.

## Discussion

SBP continues to be a highly morbid and potentially deadly condition, particularly among patients with cirrhosis and ascites. Effective management is centered around the prompt identification of the causative organism, as this informs the appropriate antibiotic therapy. Traditionally, third-generation cephalosporins like cefotaxime have been the cornerstone of initial treatment. However, the rise of antibiotic-resistant pathogens and the increasing prevalence of gram-positive organisms necessitate adjustments in empirical therapy [1,2].

SBP is the most frequent bacterial infection encountered in patients with cirrhosis, accounting for 10%-30% of all bacterial infections in hospitalized cirrhotic patients. In contrast, the prevalence among asymptomatic outpatients is relatively low, around 3.5% or less. However, this prevalence dramatically increases in the nosocomial setting, ranging from 8% to 36%, underscoring the need for vigilance in hospital environments [3].

Most SBP cases are caused by aerobic gram-negative bacteria, such as *E. coli* and *K. pneumoniae*, while aerobic gram-positive bacteria account for the remainder. Despite the abundance of anaerobes in the distal small bowel and colon, they rarely cause SBP due to the high oxygen tension in ascitic fluid, which is inhospitable for anaerobic growth [4].

*C. perfringens*, an anaerobic, gram-positive bacillus is an exceptionally rare cause of SBP, attributable to the oxygen-rich nature of ascitic fluid, which creates a harsh environment for the organism to grow. The likely infection mechanism involves the transmural migration of bacteria from the intestinal lumen. Cirrhotic patients often experience bacterial overgrowth in the small intestine due to decreased intestinal motility, and intestinal inflammation can further facilitate bacterial translocation. In cases of *C. perfringens* peritonitis, it is critical to rule out bowel perforation, as it poses a serious threat to the patient’s health [4]. Although there is no characteristic presentation of *C. perfringens *in ascitic fluid given its rarity, it could potentially be a pathogen associated with loculations, which should raise clinician suspicion for uncommon pathogens when present [5].

As illustrated in the discussed case, CT scans did not reveal any perforation, although micro-perforations could have contributed to the *C. perfringens* infection. Another potential infection route is nosocomial or iatrogenic transmission, possibly from previous paracentesis procedures performed before the current hospitalization.

This case underscores the need for continuous monitoring and potential adjustments in antibiotic therapy, reflecting the evolving microbial landscape and emerging resistance patterns. Addressing the underlying liver disease is paramount, as is improving infection control practices in healthcare settings to reduce SBP incidence and improve patient prognosis. Regular surveillance, timely diagnosis, and tailored antibiotic strategies are essential components of effective SBP management.

In the setting of improved laboratory values with empiric treatment, it is imperative to use further imaging modalities, such as US or CT to monitor the patient’s response to therapy [6]. Improved laboratory values, such as reduced white blood cell counts and normalized liver function tests, do not always correlate with complete resolution of infection or abscesses, particularly in cases with loculated ascites. Regular imaging follow-up helps in assessing the resolution of loculations, detecting any new fluid collections, and ensuring that other complications are not present, such as abscesses or bowel perforations. Recurrent ascites with worsening loculations may benefit from the use of intraabdominal fibrinolysis; however, there is limited data on its use in the literature [7].

When managing ascites with loculations in a patient with a history of multiple interventions, initial clinical suspicion and the prompt initiation of broad-spectrum antibiotic therapy are crucial to decrease morbidity and mortality. These patients are at an increased risk for complicated infections, including those caused by resistant or atypical pathogens. While the ascitic fluid is being cultured for these patients, broad-spectrum antibiotics such as vancomycin, piperacillin-tazobactam, third-generation cephalosporins, or a fluoroquinolone combination should be used to prevent progressing bacteremia [8]. Therefore, a proactive and comprehensive approach to diagnosis and treatment is essential.

## Conclusions

The patient was found to have a history of multiple interventions, such as repeated paracentesis or other invasive procedures, which increased the risk for complicated infections caused by resistant or atypical bacteria. Clinicians should maintain a high index of suspicion for complex infections, including SBP. In the presence of ascitic loculations, clinicians should consider the possibility of infection caused by less common organisms, such as *Clostridium *species. Empirical antibiotic therapy should be broad enough to cover a wide range of potential pathogens, including resistant gram-negative and gram-positive bacteria, as well as anaerobes. Initial antibiotics might include a combination of third-generation cephalosporins (e.g., cefotaxime) and metronidazole, or broader-spectrum agents like piperacillin-tazobactam, depending on local resistance patterns and patient history.

Early diagnosis and appropriate management of SBP are paramount to improving patient outcomes. Clinicians must remain vigilant and consider both common and unusual pathogens when treating patients with cirrhosis and ascites. Regular monitoring, prompt empirical antibiotic therapy, and adjustment based on culture results, along with preventive strategies in high-risk patients, are key components of an effective approach to managing this serious condition.
